# Research progress on the utilization technology of broccoli stalk, leaf resources, and the mechanism of action of its bioactive substances

**DOI:** 10.3389/fpls.2023.1138700

**Published:** 2023-03-29

**Authors:** Lu Yan, Gang Zhou, Khuram Shahzad, Haoran Zhang, Xiang Yu, Yusu Wang, Nan Yang, Mengzhi Wang, Xin Zhang

**Affiliations:** ^1^ Laboratory of Metabolic Manipulation of Herbivorous Animal Nutrition, College of Animal Science and Technology, Yangzhou University, Yangzhou, China; ^2^ State Key Laboratory of Sheep Genetic Improvement and Healthy Production, Xinjiang Academy of Agricultural Reclamation, Shihezi, China; ^3^ Huaiyin Institute of Agricultural Sciences in Xuhuai Region, Huaian, China; ^4^ Department of Biosciences, COMSATS University Islamabad, Islamabad, Pakistan

**Keywords:** broccoli, stalks and leaves, sulforaphane, kaempferol, resource utilization

## Abstract

Broccoli is a nutritious vegetable. It is high in protein, minerals, and vitamins. Also, it possesses antioxidant activities and is beneficial to the human body. Due to its active effect, broccoli is widely accepted by people in daily life. However, in terms of current utilization, only its florets are consumed as vegetables, while more than half of its stalks and leaves are not utilized. The stalks and leaves contain not only nutrients but also bioactive substances with physiologically regulating properties. Therefore research into the action and mechanism of its bioactive substances as well as its development and utilization technology will make contributions to the further promotion of its resource development and utilization. As a theoretical foundation for the resource utilization of broccoli stalks and leaves, this report will review the distribution and consumption of broccoli germplasm resources, the mechanism of action of bioactive substances, and innovative methods for their exploitation.

## 1 Introduction

Broccoli (*Brassica oleracea* var. *italica*), also called green cauliflower, is a variety of wild cabbage in the *Brassica* L (Mehraj et al., 2020). Broccoli is originally from Italy and was later introduced to England in the 18th century, with a very long history of cultivation. Known as the “vegetable crown”, broccoli has a refreshing flavor and great nutritional value, including glucoraphanin (Glu), vitamins, polyphenols and so on. Eating broccoli not only provides vital nutrients but also helps to prevent and fight against common diseases (Bessler and Djaldetti, 2018). Today, broccoli is planted widely in various countries including the east and south of China being the two primary planting regions. Broccoli is produced in huge quantities, yet the stalks and leaves are rarely utilized, resulting in a waste of resources and even environmental pollution. To improve the utilization rate of broccoli stalks and leaves, and to decrease resource waste, a series of explorations have been conducted on the bioactive substances in broccoli and the novel methods for broccoli utilization.

According to previous studies, the mainly bioactive substances in broccoli are Glu, polyphenols and flavonoids, like kaempferol (Kea). These all have antioxidant, antibacterial and anti-inflammatory properties. Sulforaphane (SFN), which is generated when Glu is hydrolyzed, also has anti-cancer properties (Sarbu et al., 2019; Biharee et al., 2020). Except the direct extraction and addition of the bioactive substances, advanced technologies have been explored for the exploitation of broccoli. For example, broccoli aptamers function as fluorescent RNA aptamers that unite fluorescent dye and generate fluorescence, providing a convenient method to visualize intrinsic RNA in cells (Chandler et al., 2018). *Candida albicans* and other bacteria are inhibited by the supernatant of fermented broccoli produced by *Bifidobacterium longum* and *Lactobacillus* (Suido and Miyao, 2008). In order to provide some reference materials for the development and utilization of broccoli stalk and leaf resources, this paper will review the distribution of broccoli resources and its stalk and leaf utilization, the types, effects, and mechanisms of action of bioactive substances, and advanced technologies for the development and utilization of broccoli.

## 2 Distribution of broccoli resources and utilization of stalks and leaves

### 2.1 Distribution of germplasm resources of broccoli

**Figure 1:**
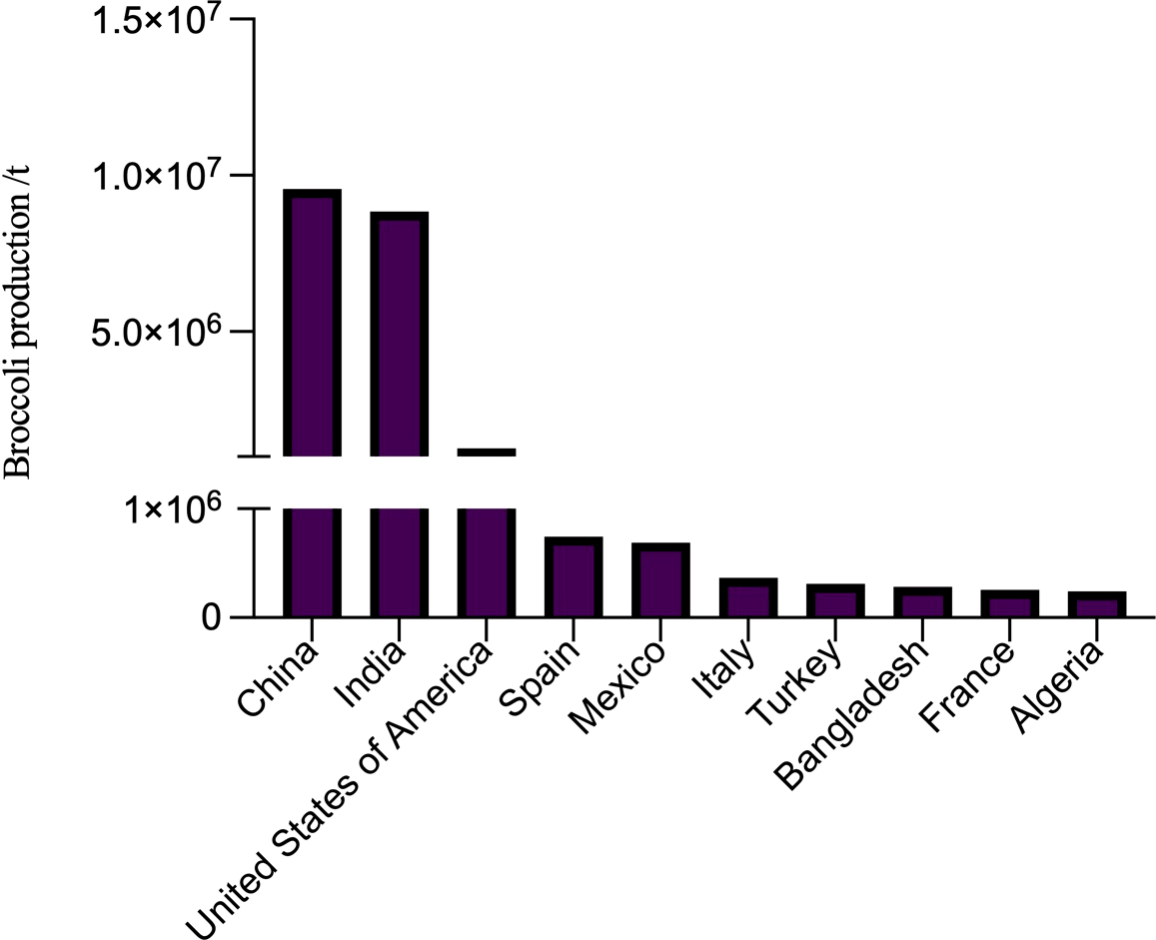
Broccoli production by different countries in 2020 (top 10).

According to the Food and Agriculture Organization of the United Nations (Food and Agriculture Organization of the United Nations, 2021), global broccoli production in 2020 is 25.53 million ton (t) including countries such as China, India and the United States having more than 1 million t. Among these countries, China has gotten the first place containing up to 9.55 million t production (**Figure 1**).

The diversity of broccoli varieties is very abundant. Shen et al. (2021) analyzed 372 broccoli varieties and discovered that there was a very large diversity among the most different varieties of broccoli, with only a tiny percentage exhibiting affinities. These 372 varieties were divided into two groups by cluster analysis, with group 1 (98) being dominated by improved varieties and group 2 (274) being dominated by local varieties. China, as the largest broccoli producer at present, showed close affinity between Chinese broccoli varieties and the core Japanese varieties (Stansell and Björkman, 2020).

### 2.2 Utilization of broccoli stalks and leaves

#### 2.2.1 Broccoli stalk and leaf production

**Figure 2:**
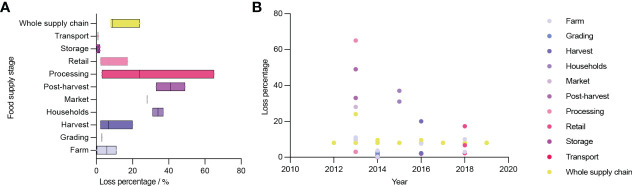
Broccoli loss percentage in different steps from 2012 to 2019. **(A)** Broccoli loss percentage in different steps. **(B)** Broccoli loss percentage in different years.

Despite the huge amount of production, the abundance of germplasm and the growing market, there are still issues related with the broccoli industry. The low utilization of broccoli stalks and leaves is among the most common, which results in considerable waste. The stalks and leaves are discarded during planting, processing, transportation and marketing for various reasons, such as the low consumption rate of stalks and leaves, underdeveloped florets, pests and diseases. In 2013, the loss rate of Australian broccoli during processing was up to 65% (**Figure 2**)(Food and Agriculture Organization of the United Nations, 2022). This causes very serious losses, especially with the large number of discarded stalks and leaves produced each year. As a result of the high water content and high perishability of these discarded stalks and leaves, improper disposal will contaminate the neighboring air and water sources, putting a great deal of strain on the ecosystem.

After analyzing the current situation of broccoli resource utilization, it can be seen that broccoli has huge losses during the production process. The resource utilization of broccoli stalks and leaves and damaged florets can promote the development of the industry in several different ways such as environmental health dimension: reduce pollution and protect the ecological environment; economic dimension: reduce costs, lower commodity prices, or increase product profits; scientific dimension: scientific research to solve the problem of resource utilization can promote scientific progress; social dimension: promote social development and respond to the concept of sustainable development.

#### 2.2.2 Broccoli stalk and leaf utilization pathway

Broccoli waste stalks and leaves can not only offer nutritional values but also can be used in feed, such as silage preparation or leaf protein powder, and can be extracted for high-value products (bioactive substances) for utilization, which helps to reduce the negative environmental impact of these wastes.

##### 2.2.2.1 Preparation of silage

Partovi et al. (2020) mixed broccoli stalks, leaves and wheat straw for silage, and verified that feeding broccoli-straw silage to fattened lambs had no negative impacts. Meneses et al. (2020) fermented raw artichokes and cooked broccoli in plastic bags for 72h to produce two kinds of silages, revealed that the *in vitro* dry matter (DM) digestibility of both silages was high, and the *in vivo* crude protein and NDF digestibility were also observed high. Both silages met the standards for plant disease residues. Monllor et al. (2020) investigated the nutritional composition of broccoli silage at various periods, and showed that stability of broccoli silage was reached at 30 days. It was suitable for ruminant feed when it reached 200 days due to its superior microbial and nutritional composition. Muelas et al. (2022) added 40% broccoli by-product silage to a balanced ration of dairy goats over a long period, and showed that the milk produced by dairy goats fed with broccoli silage was suitable for yogurt and cheese fermentation and that mixing broccoli silage with balanced ration improved the antioxidant performance of dairy goats.

##### 2.2.2.2 Preparation of leaf protein powder

Krupa-Kozak et al. (2021) added broccoli leaf powder to gluten-free bread and demonstrated a considerable increase in the protein and mineral contents of gluten-free bread. Additionally, the use of broccoli leaf powder augmented the anti-aging and antioxidant properties of gluten-free bread. Drabińska et al. (2018) investigated the effects of adding broccoli leaf powder to gluten-free sponge cakes and found that by adding 2.5% broccoli leaf powder to replace starch has increased the antioxidant activity of the new gluten-free sponge cakes. Drabińska et al. (2022) found that the optimal cooking time and water absorption were observed to be shortened when 5% broccoli leaf powder was added to durum wheat pasta, which might boost the mineral and protein contents of the pasta without altering the sensory evaluation.

##### 2.2.2.3 Extraction of bioactive substances

The main bioactive substances in broccoli stalks and leaves are Glu and polyphenols. Song et al. (2020) found that isoprenaline causes myocardial oxidative stress and that SFN treatment can increase endogenous antioxidant activity and prevent isoprenaline-induced myocardial damage. In a study of Ranaweera et al. (2022), it was found that SFN could regulate the expression of metabolism-related genes of lipid and exert an anti-obesity ability in a mouse ulcerative colitis (UC) model induced by dextran sodium sulfate (DSS). In order to alleviate the symptoms of colitis, Qu et al. (2021) discovered that Kea reduced the levels of LPS, IL-1b, IL-6 and TNF-a, increased the mRNA expression of IL-10, and improved the gut microbiota structure *via* increasing the proportion of beneficial bacteria (like *firmicutes* and *bacteroidetes*). According to Kim et al. (2018), Kea activated IRE1-JNK-CHOP signaling from the cytoplasm to the nucleus and inhibited the G9a pathway to cause autophagic death in gastric cancer cells. Ward et al. (2018) treated prostate cancer cells with different concentrations of quercetin and found that cancer cells exhibited a time- and dose-dependent reduction of cell viability while having no effect on healthy prostate epithelial cells.

Broccoli stalks and leaves can be utilized in many ways, but the main one is the extraction of its bioactive substances for the defense of various inflammatory diseases and disorders. By fusing agriculture with other industries, new waste treatment methods can be explored in the future. For example, using middle tender part of the stalk to make pickled vegetables (Zhacai) like *Brassica juncea* var. *tumida Tsen et Lee* in the genus Brassica (Zeng et al., 2022) or making the stalks into carved decorations to decorate the dishes. It is worth noticing that these agricultural wastes may produce some harmful substances or have some rotten in the process of storage and need to be assessed for biosafety in the process of utilization.

## 3 Main bioactive substances in broccoli stalks and leaves

Dietary fiber, vitamins, minerals, and bioactive substances like SFN and Kea are all abundant in broccoli. The majority of bioactive substances generated from plants have potent antibacterial and antioxidant properties (Dominguez-Perles et al., 2011). Broccoli waste stalks and leaves were determined to contain adequate bioactive substances (**Table 1**), and the high content of dietary fiber can be fed to animals to promote intestinal digestion and absorption, improve absorption rate, and prevent accumulation of food.

**Table 1:** Content of bioactive substances and nutrient in stalks and leaves of broccoli.

	Stalks	Leaves	Reference
Glucoraphanin, μmol/g DW	1.503	0.682	(Li et al., 2021)
Polyphenols, mg/g DW	9.78-11.74	99.377-135.64	(Domínguez-Perles et al., 2010)
Dietary fibres, %	–	26–32%	(Berndtsson et al., 2020)
Protein, g/100g DW Ash, g/100g DW Total lipid, g/100g DW Total carbohydrates, g/100g DW	8.76 9.24 6.58 75.42	12.13 14.67 6.72 66.48	(Campas-Baypoli et al., 2009) (Campas-Baypoli et al., 2009) (Campas-Baypoli et al., 2009) (Campas-Baypoli et al., 2009)

### 3.1 Sulforaphane

SFN, an isothiocyanate, is created when Glu is hydrolyzed in the presence of black mustard enzymes. The research team led by Professor Paul Talalay at Johns Hopkins University in the United States discovered that SFN in broccoli is a significant and powerful inducer of phase II cancer-protective enzymes while studying the cancer-preventive effects of broccoli (Zhang et al., 1992). A further study conducted by Fahey et al. (1997) uncovered that Glu, which is a precursor to SFN and is highly unstable at ambient temperature, is abundant in broccoli seeds and seedlings. There are two primary pathways for Glu conversion, which are either by the black mustard enzyme produced by broccoli or by the action of gut microbiota (Latté et al., 2011).

There is also a wide variety of glucosinolates, classified into aliphatic glucosinolates, aromatic glucosinolates and indole glucosinolates based on R side chains (Sánchez-Pujante et al., 2017). The scientific name of glucosinolate in broccoli is 4-methylsulfinylbutylthioside, also called glucoraphanin, which is an important metabolite in broccoli. The Glu is isolated from the black mustard enzyme in intact broccoli. The Glu is mainly present in the cells between the plant phloem and endodermis, whereas the black mustard enzyme is present in the vesicles of the thin-walled tissue cells of the phloem. When the broccoli tissue is mechanically damaged, resulting in cell rupture, the black mustard enzyme in the cell lysosome will be released and promote the hydrolysis of Glu to produce isothiocyanate, nitrile, SFN and other active substances(Wittstock and Halkier, 2002; Chen et al., 2021) (**Table 2**).

**Table 2:** Enzymatic hydrolysates of glucoraphanin.

Compound	Formula	Molecular weight	Relative abundance
Allyl isothiocyanate	C_4_H_5_NS	99	1.39
Isobutyl isothiocyanate	C_5_H_9_NS	115	0.32
1-butenyl isothiocyanate	C_5_H_7_NS	113	12.17
4-(Methylthio)butanenitrile	C_5_H_9_NS	115	1.59
5-(methylthio)-valeronitrile	C_6_H_11_NS	129	5.05
3-(Methylthio)propyl isothiocyanate	C_5_H_9_NS_2_	147	0.72
Butyl isothiocyanate	C_5_H_9_NS	115	4.15
Sulforaphane nitrile	C_6_H_11_NOS	145	0.22
Sulforaphane	C_6_H_11_NOS_2_	177	74.42

Various enzymatic conditions resulted in different chemicals being generated. When pH < 6.5 and epithiospecifier proteins (ESP) are present, they promote the synthesis of nitrile while inhibiting the synthesis of SFN. When 2 < pH < 5 and Fe^2+^ and nitrile-specifier proteins (NSP) are present, they promote nitrile. When pH > 8 and there is a presence of thiocyanate-forming protein (TFP), the production of isothiocyanate is favored. Only when pH is maintained in the neutral range, the production of SFN is favored (Shokri et al., 2021; Sikorska-Zimny and Beneduce, 2021).

The Glu content of different broccoli varieties varied as well. The study conducted by Traka et al. (2013) showed that the levels of glucoiberin (3MSP) and glucoraphanin (4MSB) in the varieties HG1, 1199 and 1639 were significantly higher than those in the standard varieties, with levels consistently 2.5-3 times higher as compared to the standard broccoli varieties. The Glu content of different parts of broccoli were also varied, specifically the root > flower bud > stalks and leaves, with the root containing nearly 43% of total Glu (Li et al., 2021). Even so, the stalks and leaves are also rich in Glu and can be used as a source of Glu in the case of resourceful usage of agricultural waste.

### 3.2 Polyphenols

Polyphenols are widely present in nature. The majority of polyphenols in nature have fragrant characteristics, and roughly 5% of all spices are made up of one or more of their species. In Brassica, the most prevalent polyphenols are flavonoids (mostly flavonols, a few anthocyanins) and hydroxycinnamic acid (Cartea et al., 2010). In 1930, scientists discovered a substance in citrus that was initially thought to be vitamin P, but it was later confirmed to be a flavonoid called “rutin” (Biharee et al., 2020). Polyphenols play significant roles in nature processes such as UV protection, preventing pigmentation, and maintenance of disease resistance in nature, in which they are highly concentrated in plant leaves and fruit epidermis (Cartea et al., 2010).

Based on the position of the phenyl ring, three major classes of flavonoids can be recognized: flavonoids (position C-2), isoflavonoids (position C-3) and neoflavonoids (position C-4). In addition, each group is further divided (flavones, flavonols, etc.) according to the oxidation, saturation and substituents of the pyran core. In addition to these three classes, another class of flavonoids is also considered as minor flavonoid, which includes chalcones and aurones, without benzopyran (Sarbu et al., 2019).

The primary flavonoids in Brassica crops are flavonols, which are a subgroup of predominantly 3-hydroxyflavonoids, including quercetin, Kea and isorhamnetin. Conjugation occurs most often at the C-3 position, but substitution can also occur at the 5, 7, 4’, 3’ and 5’ positions. These are mainly bound to glucose and commonly acylated with different hydroxycinnamic acids (Cartea et al., 2010). In the experiments of Duan et al. (2021), 15 different varieties of broccoli were selected, in which the accumulation of Kea in leaves was greater than that of quercetin, and in some varieties the accumulation of Kea in florets was greater than that of quercetin, so the most dominant flavonol in broccoli stalks and leaves was Kea, and the study also indicates that the content of flavonols in leaves was higher than that in florets. In conclusion, broccoli stalks and leaves are able to be a high-quality source of Kea.

In addition, broccoli has other nutrients such as vitamins (Va & Vc), minerals (Ca, P & Fe), phenolic acids, etc. SFN and Kea are the two bioactive substances that have been most intensively studied in previous studies. In future studies, other bioactive substances and their metabolites in broccoli stalks and leaves, as well as their combined effects can also be explored.

## 4 The main bioactive substances of broccoli stalks and leaves with their mechanism of action

### 4.1 Sulforaphane

#### 4.1.1 Anti-cancer

In cancer cells, the content of glutathione (GSH) is higher than healthy cells. GSH concentrations in cancer cells are more than 1,000 times higher than extracellular fluid and four times higher than in healthy cells, particularly in drug-resistant tumor cells, where these are even ten times higher. (Kalinina and Gavriliuk, 2020). SFN and GSH in cancer cells generate SFN-GSH by the action of glutathione S-transferase (GST). SFN-GSH generates SFN-Cys-Gly, SFN-Cys and SFN-NAC *via* the action of γ-glutamyltranspeptidase, cysteinylglycinase and N-acetyltransferase. As soon as SFN enters the cell, it rapidly interacts with GSH to form SFN-GSH, leading to its accumulation in the cell. Due to the high GSH content of cancer cells, the accumulation of SFN was encouraged, and the anti-cancer effect of SFN was enhanced (Gu et al., 2022).

The way SFN exerts its anti-cancer effect is mainly manifested by its inhibitory effect on histone deacetylase (HDAC). According to their sequence homology, four classes of HDAC are divided: I HDAC (1, 2, 3 and 8); II HDAC: IIa (4, 5, 7 and 9) and IIb (6 and 10); III HDAC (sir2); and IV HDAC11 (de Ruijter et al., 2003). Several studies (Chen et al., 2014; Jiang et al., 2016; Bär et al., 2022) have indicated that SFN inhibits HDAC to prevent cell proliferation and induce apoptosis, leading to the downregulation of E2F3 and Ki-67, and activation of p21, bax and caspase-3 in cancer cells.

**Figure 3:**
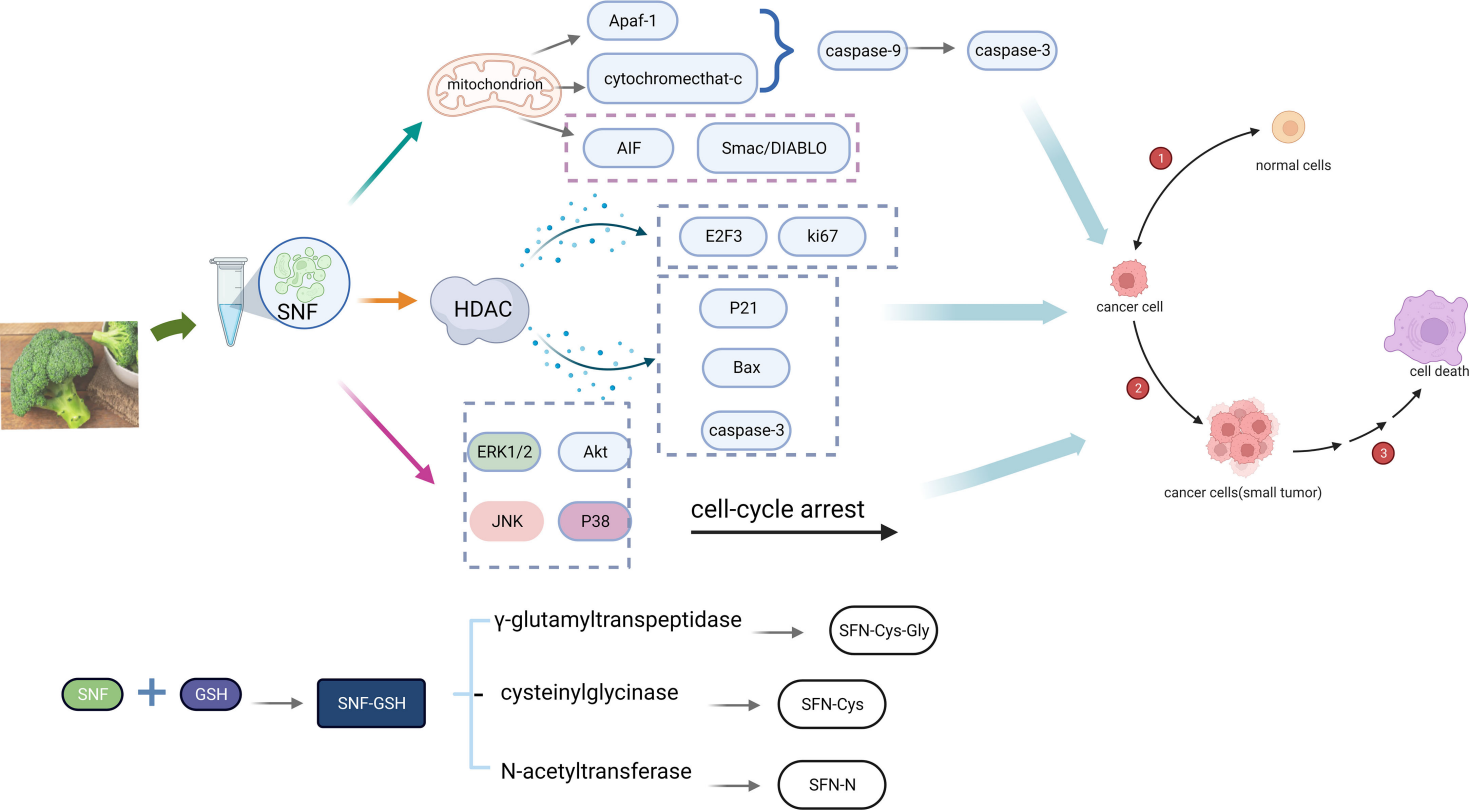
Mechanism of anti-cancer action of SFN (Created with BioRender.com).

SFN (20-40 μM) disrupting the mitochondrial membrane potential to induce apoptosis in many types of cancer cells, with mitochondrial release of cytochrome c, Smac/DIABLO and AIF. SFN (40 μM) mediates apoptosis by activating ERK1/2 and protein kinase B (protein kinase B, Akt), causing cell cycle arrest and death (Choi and Singh, 2005). Further studies (Rudolf et al., 2009) also have shown that JNK and p38 are two ways that SFN might cause apoptosis. It has also been demonstrated that SFN is able to disrupt the endoplasmic reticulum and then successively activate the effector proteins calpain, caspase-12, caspase-9, and caspase-3 (De Gianni and Fimognari, 2015) (see **Figure 3**).

In addition, Elkashty and Tran (2020) investigated whether SFN enhances the chemotherapeutic effect of cis-chloroplatinum (CIS) and the inhibitory effect of 5-fluorouracil (5-FU) on head and neck squamous cell carcinoma-cancer stem cells (HNSCC-CSC). They found that SFN reduced the viability of drug-resistant HNSCC-CSC with time and dose, and the combination of SFN increased their cytotoxicity (CIS by 2-fold, 5-FU by 10-fold). Fortunately, it has no effect on the activity and function of non-cancerous stem cells.

Although SFN has anti-cancer effects, it is far from being able to cure cancer and should only be used as a daily cancer preventative. Future research could look into combining SFN with other treatments to lessen the negative effects of chemotherapy on the body.

#### 4.1.2 Antioxidant and antibacterial

SFN stimulates the trophic genome of Nrf2 to become active (Zhang et al., 2018). All human organs contain Nrf2, a transcription factor that is crucial for endogenous protection against oxidative stress and defense against external harmful chemicals (He et al., 2020a). The expression of genes with cytoprotective properties is controlled by Nrf2 activation, and this protects the body by regulating the production of proteins from these genes that have anti-oxidant, anti-inflammatory, anti-glycation, and other protective properties. (He et al., 2020b).

SFN has antibacterial properties and can protect plants from pathogenic bacteria and phytopathogens. SFN inhibits bacteria and fungi such as *Escherichia coli*, and exerts antimicrobial effects *in vitro* against foodborne pathogens and enteropathogenic microorganisms, including *Listeria monocytogenes*, *Escherichia coli*, *Salmonella typhimurium* and *Helicobacter pylori* (Abukhabta et al., 2021). In future perspectives, it can function as an additive to bacterial isolation and purification media, or be combined with beneficial bacteria to produce probiotics used to improve gut microbiota structure.

### 4.2 Kaempferol

#### 4.2.1 Antioxidant

Oxidative stress is a state of imbalance between oxidants and antioxidants that lead to incomplete degradation of reactive oxygen species (ROS) and reactive nitrogen species (RNS) in cells (Sies, 2015). ROS interacts with biomolecules, leading to damage to proteins, lipids and DNA molecules while drawing inflammatory mediators that stimulate inflammatory mechanisms. Inflammatory cells, such as neutrophils and macrophages, produce ROS accompanied by superoxide anion production, leading to cellular damage (Sova and Saso, 2020).

Kea has good antioxidant activity and can react cell-free *in vitro* with H_2_O_2_, HOCl, superoxide and NO. Kea also modulates the awakening of the redox-sensitive transcription factor Nrf-2, which in turn promotes the activation of heme oxygenase-1 (HO-1) and shields cells from oxidative damage (Hu et al., 2021).

#### 4.2.2 Anti-inflammatory

Phospholipase A2 (PLA2) and lipoxygenase (LOX) control the production of prostaglandins (PG), which are inflammatory mediators produced by cyclooxygenase (COX) through the metabolism of arachidonic acid (AA) in the inflamed tissues (Yahfoufi et al., 2018). Kea has a significant inhibitory effect on COX-1, COX-2, and LOX (Lee and Kim, 2010). When a large amount of NO is present, PG production is further amplified, which is accelerated by the combined expression of inducible NO synthase (iNOS) and COX-2 in inflamed tissues, resulting in increased PG production (Wu, 2006). The expression of iNOS determines the physiological and pathological effects of NO, where an excess of NO can cause a number of clinical problems, particularly inflammation (Kielbik et al., 2019). NO interacts with transition metal ions and modifies the activity of various enzymes, such as catalase, leading to the accumulation and toxicity of H_2_O_2_. In addition, NO interacts with superoxide anions to form peroxynitrite, which functions as a strong oxidant, causing LDL oxidation, DNA damage, inhibition of mitochondrial respiration, and apoptosis. NO has also been shown to induce the tumor necrosis factor TNF-α (Park et al., 2012). Kea inhibits lipopolysaccharide (LPS) induced NO production and also reduces TNF-α, thereby decreasing the inflammatory response(Bian et al., 2020).

**Figure 4:**
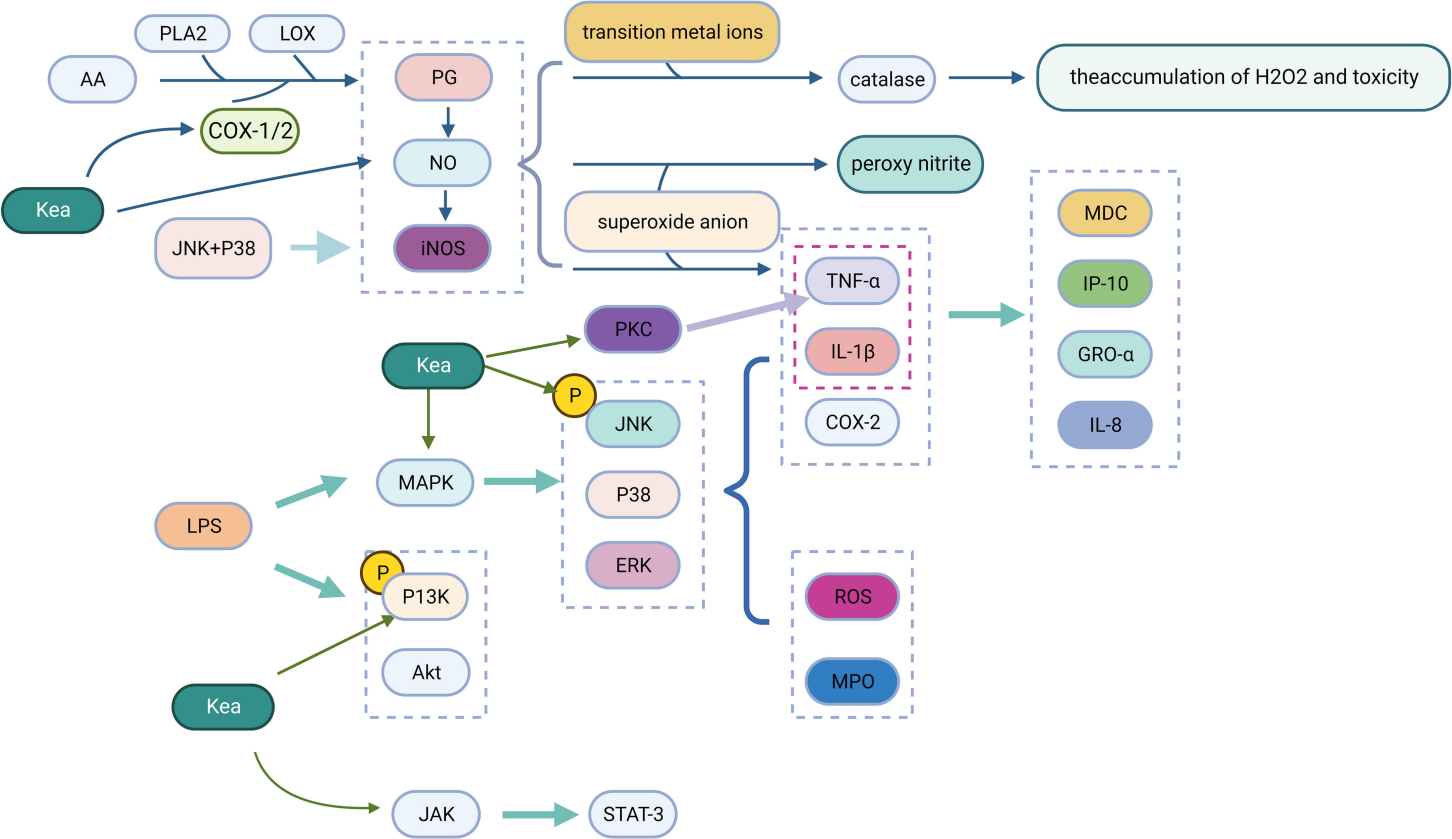
Anti-inflammatory mechanism of Kea (Created with BioRender.com).

Mitogen-activated protein kinases (MAPK), protein kinase C (PKC), phosphatidylinositol 3 kinase (PI3K) and the JAK-STAT signaling pathway are involved in regulating the expression of inflammatory mediators, the transcription and expression of various transcription factors (Hu et al., 2001). MAPK includes extracellular signal-regulated kinases 1/2 (ERK1/2), c-jun N-terminal kinases (JNK) and p38 mitogen-activated protein kinases (p38) (Kim and Choi, 2010). This pathway is activated in response to inflammatory stimuli, which causes the expression of target genes (TNF-α, IL-1β, COX-2 and collagenase). Kea blocks the expression of the LPS-induced MAPK pathway, which reduces the inflammatory burden by suppressing the synthesis of IL-8, the interferon-induced protein (IP-10), the growth-related oncogene (GRO-α) and the macrophage-derived chemokine (MDC). Kea inhibits JNK and p38 phosphorylation induced by LPS and IL-1α, which are responsible for the synthesis of NO, PGE2 and iNOS expression. When LPS causes lung damage in BALB/c mice, the phosphorylation of ERK, p38, and JNK is activated, which regulates the production of myeloperoxidase (MPO), ROS, and pro-inflammatory mediators, where Kea can inhibit ERK, p38 and JNK pathway activation to mitigate their adverse effects (Bian et al., 2020). Kea inhibited LPS-induced PI3K and Akt phosphorylation in mouse A549 cells, cardiac fibroblasts and microglia BV2 cells, reduced the production of RANTES, and protected cells from inflammatory factor activation (Park et al., 2011). After various stimuli, the JAK-STAT (janus kinase, JAK; signal transducer and activator of transcription, STAT) pathway is abnormally activated, leading to the activation of genes encoding inflammatory mediators. Kea significantly inhibits LPS-induced inflammatory responses by blocking the JAK-STAT signaling pathway and effectively interfering with the transcriptional activation of the signal transduction, and transcriptional activation protein STAT-3 at the same time to inhibit further activation of inflammatory cytokines (Devi et al., 2015) (**Figure 4**).

The antimicrobial activity of plant-derived flavonoids exhibits a different mechanism from that of traditional medications. Therefore, bacteria or other pathogens are not susceptible to resistance because resistance genes do not initially encode the majority of natural compounds (Wen et al., 2020), and they may be important in enhancing antimicrobial therapy. More in-depth studies could lead to better utilization of phytogenic bioactive substances as additives in animal production practices.

Although these studies have proven that inflammation can be mitigated by these bioactive substances, in most cases, the inflammation that occurs during the breeding process is addressed with medication. It can be used as a daily inflammation prevention to reduce the likelihood of inflammation occurring throughout the breeding process and thus reduce the financial losses of the farmers. Future studies can examine whether these two main substances interact favorably or negatively with other substances and whether they can be used as additives in animal husbandry.

More diverse animal experiments are required to confirm the possibility of using broccoli bioactive substances as a medicine because the scope of animal studies on the mechanism of action of broccoli bioactive substances is relatively small.

## 5 New technology for broccoli utilization

### 5.1 Broccoli aptamer as a fluorescent RNA aptamer

Elect RNA aptamers bind to fluorescent dyes and activate fluorescence, providing a straightforward method for visualizing the original RNA within the cell.

Nucleic acid nanoparticles (NANPs) are a highly versatile molecular platform for the targeted delivery of a variety of therapeutic agents. Immunomodulation of NANPs can support both their immunostatic delivery and the conditioned stimulation of beneficial immune responses (Johnson et al., 2021). DFHBI is a small molecule that resembles the green fluorescent protein (GFP) chromophore. When unbound, spinach aptamers and DFHBI are largely nonfluorescent, yet *in vitro* and in living cells, spinach-DFHBI complexes are brilliantly fluorescent. DFHBI can be selected for imaging RNA (Santra et al., 2019).

In nucleic acid nanotechnology, any fluorescent aptamer can be readily embedded in the structure of NANPs as a functional unit by a simple extension of a single strand, and the responsive behavior of NANPs can be directly observed in living cells using dye-labeled pairs of complementary strands. The high cytotoxicity of the first developed malachite green (MG) aptamer and its non-specific binding to cellular substances called for new biocompatible RNA aptamers. Subsequently a spinach aptamer showed green fluorescence in combination with the GFP fluorophore analogue DFHBI, but it was susceptible to endonuclease and cellular activity. In recent years, a new broccoli aptamer named F30-Broccoli was developed, which has a three-way linkage scaffold with higher binding affinity for the ligand DFHBI-1T (Chandler et al., 2018).

### 5.2 Fermentation of broccoli supernatant by Bifidobacterium longum and Lactobacillus inhibited Candida albicans

Bifidobacterium, a non-pathogenic Gram-negative bacterium, is an important member of the human intestinal flora and inhibits the proliferation of disease-causing microorganisms by lowering the pH of the intestine through fermentation of acetic acid or lactic acid. *B. longum BB536* or *L. pentosus Vege-Start* were added to broccoli medium, cultured in an anaerobic environment, then centrifuged and filtered to obtain *B. longum* fermented broccoli (BFB) and *L. pentosus* fermented broccoli (LFB). They were found to have inhibitory activities against *Candida albicans* (Suido and Miyao, 2008).

### 5.3 Establishment of novel epigallocatechin gallate (EGCG) carriers

It has been shown that epigallocatechin gallate (EGCG) has low bioavailability (Shi et al., 2020). The glass digestive system collected more EGCG from broccoli-based EGCG than it did from EGCG alone when the same level was added to various matrices. The level of EGCG was favorably associated with the antioxidant capacity of the chyme. The carriers that are anticipated to convey and stabilize EGCG through the digestive process are broccoli carriers. (Shi et al., 2019).

## 6 Conclusion

Broccoli is widely grown and its high loss rate is an obstacle to both environmental protection and industrial development. Therefore, research on the utilization of broccoli stalks and leaves is worthy of attention. Numerous studies have shown that the bioactive substances in broccoli, such as glucoraphanin and polyphenols, have antioxidant, anti-inflammatory, antibacterial and anti-cancer properties, and can be extracted and used to reduce resource waste. In addition to extraction and addition, new utilization techniques have also been described in recent studies. However, research on the bioactive substances of broccoli has mainly centered on the role and mechanism of action of sulforaphane so far, and the research on the utilization of other active substances of broccoli or the utilization of sulforaphane mixed with other substances is still relatively limited. Under the premise of ensuring biosafety, future research can be conducted on the development of other active substances that are not commonly used in broccoli, such as nitrile and other isothiocyanates produced by the hydrolysis of glucoraphanin, or mixing broccoli bioactive substances with other active substances and beneficial bacteria for animal breeding, so that broccoli waste can be used more comprehensively. The complete broccoli stalk and leaf processing chain needs to be designed. Finally, cost control is also very important. The cost of extracting bioactive substances in the laboratory is very high, and if we want to use broccoli bioactive substances as additives in actual animal breeding, we need to consider reducing the cost in order to expand the usage. Therefore, the extraction method of the bioactive substance needs to be optimized while maintaining the quality of the extract.

## Author contributions

LY, MW, and GZ contributed to the conception of the review. LY and XY searched the database. HZ drew the figures. LY wrote the first draft of the manuscript. LY, GZ, YW, XZ, and NY made revisions to the manuscript. KS revised the manuscript language expression. All authors contributed to the article and approved the submitted version.
